# Iron regulatory proteins: players or pawns in ferroptosis and cancer?

**DOI:** 10.3389/fmolb.2023.1229710

**Published:** 2023-06-29

**Authors:** Cameron J. Cardona, McKale R. Montgomery

**Affiliations:** Department of Nutritional Sciences, Oklahoma State University, Stillwater, OK, United States

**Keywords:** iron homeostasis, cancer, iron-sulfur cluster biogenesis, programmed cell death, reactive oxygen species, lipid peroxidation

## Abstract

Cells require iron for essential functions like energy production and signaling. However, iron can also engage in free radical formation and promote cell proliferation thereby contributing to both tumor initiation and growth. Thus, the amount of iron within the body and in individual cells is tightly regulated. At the cellular level, iron homeostasis is maintained post-transcriptionally by iron regulatory proteins (IRPs). Ferroptosis is an iron-dependent form of programmed cell death with vast chemotherapeutic potential, yet while IRP-dependent targets have established roles in ferroptosis, our understanding of the contributions of IRPs themselves is still in its infancy. In this review, we present the growing circumstantial evidence suggesting that IRPs play critical roles in the adaptive response to ferroptosis and ferroptotic cell death and describe how this knowledge can be leveraged to target neoplastic iron dysregulation more effectively.

## 1 Introduction

Iron is an essential metal for all forms of life; in fact, a large portion of the Earth itself is made of iron, making it the most abundant element on the planet (Sheftel et al., 2012). The essentiality of iron has led to its long-established role in medicine. Since the 1500s, iron has been used by physicians to treat a wide range of medical ailments (Beutler, 2002). Iron primarily exists in two forms: a reduced ferrous (Fe^2+^) form and an oxidized ferric (Fe^3+^) form. In its ferrous form, iron is highly reactive, interacting with hydrogen peroxide to form reactive oxygen species (ROS) and ferric iron (Vogt et al., 2021). The importance of iron homeostasis in the prevention of human disease is well recognized and is exemplified by paitents with the hereditary iron overload disorder hemochromatosis. Individuals with hemochromatosis are at increased risk for diabetes, hematologic malignancies, colorectal and gastric cancers, as well as cirrhosis and hepatocellular carcinoma, which account for nearly 20%–30% of deaths for patients with untreated or poorly controlled hemochromatosis (Bradbear et al., 1985; Nelson et al., 1995; Pietrangelo, 2010).

Despite this potential danger, iron is necessary for DNA synthesis, cell signaling, and cellular respiration, among other functions (Vogt et al., 2021), so insufficient iron availability is also very deleterious (Camaschella, 2019). Iron deficiency is primarily caused by inadequate dietary intake, but can be secondary to infection, inflammation, and genetic disorders. The primary symptoms of iron deficiency are fatigue and reduced work capacity, but when severe, iron deficiency is also associated with impaired cognititive development and increased risk of child and maternal mortality and is a major cause of disability worldwide (Kassebaum et al., 2014; Camaschella, 2019; The Lancet, 2019). As such, both systemic and cellular iron homeostasis need to be tightly regulated to maintain health and prevent disease (Chifman et al., 2014).

Absorption of iron is regulated via secretion of the hormone hepcidin (Meynard et al., 2014) and cellular iron levels are mediated post-transcriptionally by iron regulatory proteins (IRPs) (Chifman et al., 2014). Hepcidin maintains systemic iron homeostasis by controlling the movement of iron from the enterocyte into circulation (Meynard et al., 2014). IRPs function by sensing intracellular iron levels and binding to iron responsive elements (IRE) in the untranslated regions (UTRs) of the mRNA encoding many of the proteins involved in cellular iron homeostasis (Anderson et al., 2012). Depending on the location of the IRE, IRP binding can have two vastly different effects (Anderson et al., 2012). Binding of IRPs to IREs in the 5′ UTR results in translation inhibition and binding in the 3’ UTR results in mRNA stabilization (Wallander et al., 2006).

Ferroptosis is a form of iron-dependent cell death, that is, the result of excess lipid peroxidation (Dixon et al., 2012; Stockwell, 2022). Under normal conditions, endogenous antioxidants such as Glutathione peroxidase 4 (GPX4) can alleviate these lipid peroxides (Lee et al., 2021; Stockwell, 2022). Cysteine is imported into the cell via solute carrier family 7 member 11 (SLC7A11), one half of the antiporter system Xc^−^ (Stockwell, 2022). This cysteine is then converted to cystine prior to its incorporation into glutathione (GSH) (Stockwell, 2022). GPX4 then oxidizes GSH to reduce lipid peroxides to lipid alcohols (Stockwell, 2022). Disruption of any part of these endogenous antioxidant regulatory systems is sufficient to trigger ferroptosis (Lee et al., 2021).

Oxidation in ferroptosis can occur via both iron-based auto-oxidation or enzymatic-mediated mechanisms (Lee et al., 2021). Non-enzymatic auto-oxidation is the result of Fenton-like chemistry, in which ferrous (Fe^2+^) iron reacts directly with oxygen leading to the formation of ferric (Fe^3+^) iron and a radical (Dixon et al., 2012; Lee et al., 2021). Multiple iron-containing oxidation enzymes can also lead to the development of ROS (Lee et al., 2021). However, the role of iron metabolism in ferroptosis has only just begun to be elucidated and the roles of many iron-related proteins have yet to be described. While increased mitochondrial iron import (Yuan et al., 2016) and inhibition of iron sulfur cluster biogenesis have been shown to increase ferroptosis sensitivity in cancer cells (Novera et al., 2020), cellular iron accumulation is associated with resistance to ferroptosis in neuronal and senescent cell types (Wang et al., 2016; Masaldan et al., 2018). These findings indicate that the role of iron in ferroptosis is complex and likely context- and cell-type dependent.

## 2 Dietary iron metabolism and systemic iron homeostasis

In the diet, iron is present as both heme and non-heme iron (Abbaspour et al., 2014). Non-heme iron is primarily found in plant sources, while heme iron comes directly from the myoglobin and hemoglobin in animal products (Abbaspour et al., 2014). These two forms of iron have drastically different bioavailabilities, with maximum bioavailabilities of 10 and 30-percent, respectively (Skolmowska and Głąbska, 2019). Although there is no regulated pathway for the excretion of iron, absorption of at least 1–2 mg of iron daily by enterocytes in the duodenum is necessary to directly replace iron lost through the death of skin and intestinal cells, sweat, and menstruation (Wallander et al., 2006; Anderson et al., 2012; Chifman et al., 2014). Thus, the recommended dietary allowance for iron in male and female individuals is set at 8 mg/day and 18 mg/day, respectively, to account for low bioavailability and increased loss in females.

Elemental iron from non-heme sources enters the enterocyte through solute carrier family 11 member 2 (SLC11A2, also known as divalent metal transporter I (DMT1)), a transport protein, that is, able to transport ferrous iron (Yanatori and Kishi, 2019). However, at this point dietary non-heme iron is in the ferric form, so it has to be reduced by the ferric reductase, cytochrome b reductase 1 (CYBRD1) prior to its absorption (Chifman et al., 2014). Though heme iron transporters have been described, the primary dietary heme iron transporter has yet to be identified (Muckenthaler et al., 2017). Inside the cell, iron is released from heme via the action of heme oxygenase 1 (HMOX1) and joins non-heme iron in the labile iron pool (Chifman et al., 2014). This iron can now be used by the enterocytes (Abbaspour et al., 2014), stored in the iron storage protein, ferritin (Chifman et al., 2014; Plays et al., 2021) or exported to the rest of the body via solute carrier family 40 member 1 (SLC40A1), also known as ferroportin (Sheftel et al., 2012). Because ferroportin only exports ferric iron, ferrous iron must first be oxidized by hephaestin (HEPH), a membrane-anchored multicopper ferroxidase (Deshpande et al., 2017). After movement through SLC40A1, iron binds the iron transport protein transferrin (TF), enters the plasma, and is transported to other cells throughout the body (Chifman et al., 2014). When iron levels are adequate, hepcidin antimicrobial peptide (HAMP), commonly referred to as hepcidin, is excreted by the liver and blocks the export of iron by promoting SLC40A1 internalization and degradation (Meynard et al., 2014). Due to the short lifespan of enterocytes, the remaining iron is inevitably lost when these cells are sloughed off and excreted in the feces (Anderson et al., 2012).

Two major destinations for TF-bound iron leaving the small intestine are the liver and bone marrow (Chifman et al., 2014; Meynard et al., 2014). The liver has two major iron-related functions—Iron storage and regulation of systemic iron homeostasis via HAMP (Meynard et al., 2014). Hepatocytes internalize iron via the proteins transferrin receptor and transferrin receptor 2 (TFRC, TFR2) and the homeostatic iron regulator (HFE) (Chifman et al., 2014). Transcription of hepcidin is regulated through a process in which bone-morphogenic protein six (BMP6) binds its receptor, triggering the phosphorylation of receptor mediated SMAD homolog (R-SMAD) (Meynard et al., 2014). This results in SMAD family members 1, 5 and 8 (SMAD1, SMAD5, SMAD8), forming a complex with SMAD 4, resulting in downstream suppression of hepcidin secretion (Meynard et al., 2014; Xiao et al., 2020; Xu et al., 2021). Hepcidin is primarily regulated in response to iron availability, but can also be triggered by inflammation, hypoxia and the rate of erythrocyte formation (Chifman et al., 2014). Hepatic iron stores can vary significantly based on gender and a variety of other factors (Pietrangelo, 2016). Because of its role in iron storage, multiple diseases occur due to the storage of excess iron in the liver (Pietrangelo, 2016). Although symptoms of these diseases are similar, they are the result of a variety of pathophysiologies, including genetic or acquired loss of hepcidin, inhibition of hepcidin function and loss of ferroportin (Pietrangelo, 2016).

**FIGURE 1:**
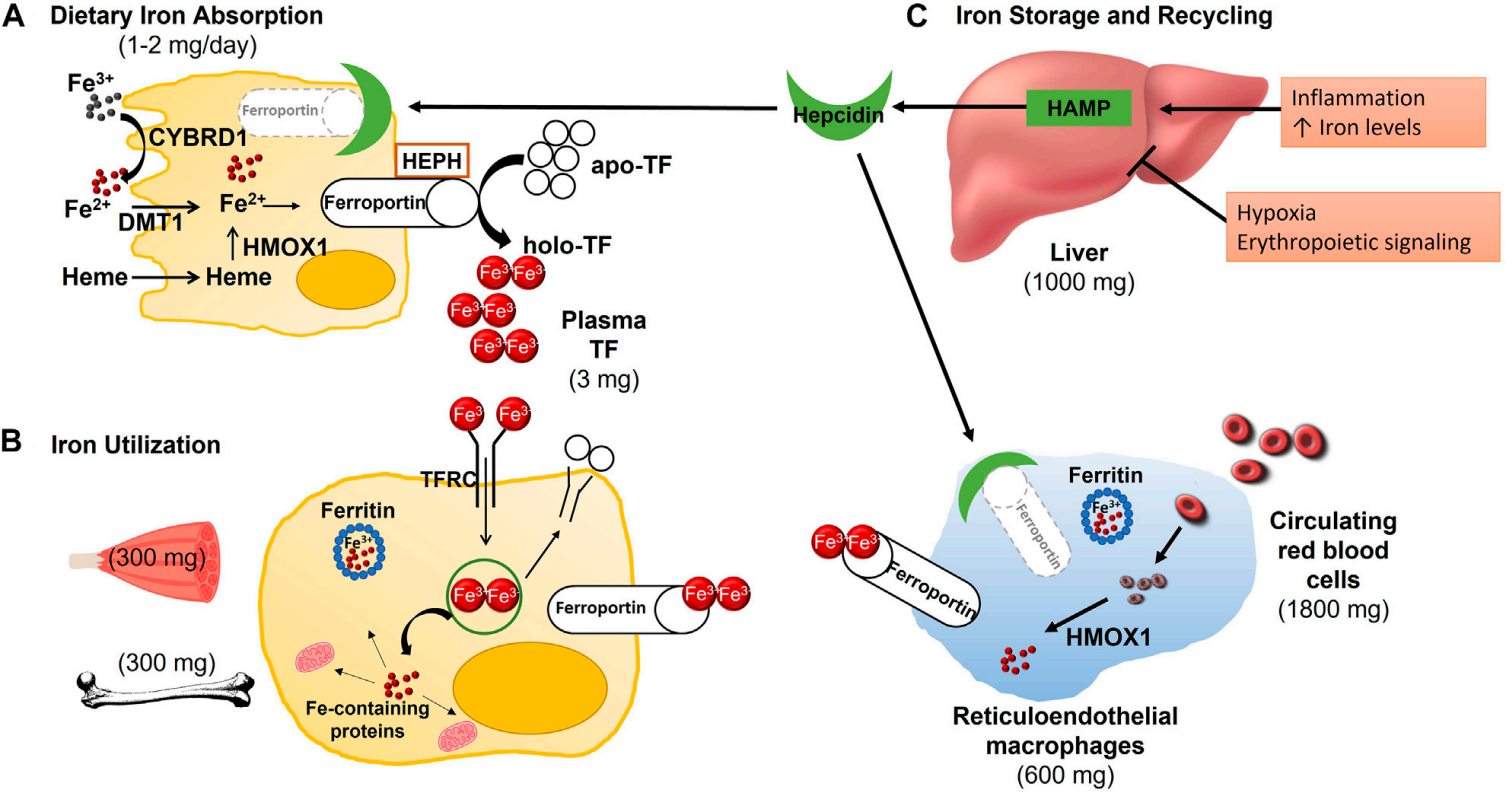
Absorption, Distribution and Metabolism of Iron. **(A)** Each day, 1–2 mg of iron are absorbed in the duodenum. Ferric, non-heme iron must first be reduced by duodenal cytochrome B (CYBRD1) to be transported into the enterocyte via divalent metal transporter 1 (DMT1). Dietary heme iron enters the enterocyte via an unknown transporter where the iron is then released from heme by heme oxygenase (HMOX1), joining non-heme iron in the labile iron pool. If not needed by the enterocyte, the ferrous iron is released into circulation, by exiting the cell through ferroportin. Following export across the basolateral membrane, ferrous iron is oxidized by hephaestin (HEPH) before it is bound to transferrin (TF) for transport to various tissues. **(B)** Transferrin-bound iron binds to transferrin receptor (TFRC) on the cell surface where the TF/TFRC complex is internalized though receptor-mediated endocytosis. The acidic pH of the endosome results in the release of iron from TF so that it can be pumped into the cytoplasm, most likely through DMT1. TF and TFRC are recycled back to the cell surface where they dissociate upon encountering a neutral pH. Inside the cell, iron can be stored in ferritin, utilized for iron-dependent processes such as the synthesis of myoglobin in skeletal muscle or erythropoiesis in the bone marrow, or exported back into circulation via ferroportin. **(C)** Whole-body iron homeostasis is maintained though the hepatic synthesis of hepcidin (HAMP) and the efficient recycling of senescent red blood cells by reticuloendothelial macrophages. In response to inflammation and increased iron availability, hepcidin production is increased. The binding of hepcidin to ferroportin at the cell surface of enterocytes and macrophages leads to its internalization and degradation, subsequently diminishing iron absorption and release from stores, respectively. Hepcidin production is decreased in response to hypoxia and enhanced erythropoiesis to increase iron uptake and availability.

Another major destination for iron in the body is the erythroid bone marrow (Chifman et al., 2014). Here, iron is essential for erythropoiesis as it is a major component of hemoglobin (Kautz and Nemeth, 2014). Iron enters erythroblasts via TFRC-mediated endocytosis and the mitochondria through the protein solute carrier family 25 member 37 (SLC25A37), also known as mitoferrin-1 (Chifman et al., 2014). In both the cytosol and mitochondria, it is used for heme formation, prior to being combined with the globin chains synthesized in the cytoplasm to form hemoglobin (Anderson et al., 2012; Chung et al., 2012; Farid et al., 2022). Once mature erythrocytes become senescent, their iron can be recycled by macrophages of the reticuloendothelial system (RES) (Chifman et al., 2014). RES macrophages phagocytose erythrocytes forming an erythrophagolysosome (EPL) that travels through the cytoplasm to the endoplasmic reticulum (ER) (Sheftel et al., 2012). The ER is then able to recruit HMOX1 (Sheftel et al., 2012) which frees iron for use by the macrophage or export to other cells through ferroportin (Chifman et al., 2014). This system is well described; however, the exact mechanism by which iron leaves the EPL has yet to be elucidated (Sheftel et al., 2012). Most iron used by the body is maintained through this efficient iron recycling system involving the bone marrow, erythroblasts, and reticuloendothelial macrophages (Winn et al., 2020). Figure 1 illustrates how iron status is sensed by the liver to regulate iron absorption, recycling, and distribution throughout the body.

## 3 IRP-mediated control of cellular iron metabolism and homeostasis

Across the body, the movement of iron into and out of cells must be tightly regulated. Cellular iron metabolism and homeostasis are regulated by the binding of iron regulatory proteins (IRPs), aconitase 1 (ACO1, also known as IRP1) and iron response element binding protein 2 (IREB2, also known as IRP2) to iron-responsive elements (IRE) in the untranslated regions (UTRs) of the mRNA of many iron related proteins (Anderson et al., 2012). The IRE is a stem looped portion of the mRNA containing the sequence CAGUG followed by uracil or cytosine, that is, around 28 nucleotides in length (Wallander et al., 2006). This region is highly conserved across IRE containing mRNAs (Wallander et al., 2006). IREs can be located in either the 3′ or 5′ UTRs of mRNA, with 5′IRE-IRP binding resulting in translational repression, and 3′IRE-IRP binding inhibiting endonucleolytic degradation (Wallander et al., 2006; Anderson et al., 2012). Classic examples of 5′IRE containing mRNAs include those involved in the storage (FTH1, FTL) and export (SLC40A1) of iron and examples of 3′IRE containing mRNAs include those involved in the import (TFRC, SLC11A2) of iron (Chifman et al., 2014). In 2011, Sanchez and colleagues (Sanchez et al., 2011) identified over 30 additional IRE containing mRNAs using immunoselection and microarrays.

Although IRP1 and IRP2 are similar in structure, they do have significant differences in the way they sense iron and bind to IREs (Volz, 2008). IRP1 exists in two forms: a cytosolic aconitase isoform that doesn’t bind IREs, or an RNA binding form, that binds IREs with a high affinity (Anderson et al., 2012). In conditions where adequate iron is available, assembly of an Fe-S cluster confers enzymatic activity, whereas under low iron conditions, disassembly of the Fe-S cluster promotes IRE binding (Wallander et al., 2006). IRP1 is also able to respond to multiple non-iron inputs including the presence of reactive oxygen and nitrogen species, the presence of heme compounds and phosphorylation of the serine it the 138th position (Volz, 2008; Anderson et al., 2012), all of which result in the disassembly of the Fe-S cluster, allowing for IRE binding (Volz, 2008). In addition to these multiple feedback loops, the ubiquitin E3 ligase responsible for iron-induced proteasomal degradation of IRP2 has been shown to also ubiquitinate IRP1 (Anderson et al., 2012).

Despite having almost sixty-percent similarity to IRP1, IRP2 lacks an Fe-S cluster and is primarily regulated by alterations in protein stability (Volz, 2008). IRP2’s degradation occurs in response to ubiquitination by its E3 ubiquitin ligase, F-box and leucin-rich repeat protein 5 (FBLX5), a protein with a hemerythrin-like domain in its N-terminal that allows it to sense the presence of iron and oxygen in the cell (Ruiz and Bruick, 2014). During times where iron is limiting or the occurrence of hypoxia, the hemerythrin-like domain conformationally changes FBXL5, resulting in increased stability and IRP2-IRE binding activity (Ruiz and Bruick, 2014). IRP2 also differs from IRP1 in that it has a 73 amino acid sequence rich in cysteine, glycine, lysine, and proline (Volz, 2008). Residues in this domain can be oxidized by heme, allowing the protein RANBP2-type and C3HC4-type zinc finger containing 1 [RBCK1, also known as heme-oxidized IRP2 ubiquitin ligase (HOIL-1)] to mark it for degradation in response to heme availability (Anderson et al., 2012). Additionally, IRP2 has been shown to have altered IRE binding affinity at different stages in the cell cycle as phosphorylation at the 157th position during the G2/M transition results in an inability to bind IREs (Wallander et al., 2008).

In response to low iron, the IRE binding activity of both IRP1 and IRP2 are increased, leading to ferritin degradation and TFRC stabilization in an attempt to increase cellular iron content (Anderson et al., 2012). Circulating transferrin bound to iron is then able to bind to its receptor, found on the cell surface, resulting in receptor mediated endocytosis, during which a clathrin coated sorting endosome is formed (Sheftel et al., 2012). This endosome contains a v-ATPase pump, that is, able to manipulate the pH of the endosome, resulting in a pH of around 5.6 (Sheftel et al., 2012; Ogun and Adeyinka, 2022). At this low pH, iron is released from transferrin (Sheftel et al., 2012), allowing both TF and TFRC to return to the cell surface (Chifman et al., 2014). Within the endosome, ferric iron is reduced back to its ferrous form by a member of the six transmembrane epithelial antigen of the prostate (STEAP) family prior to its export into the cytoplasmic labile iron pool through DMT1, where it is made available to the cell or stored in ferritin (Sheftel et al., 2012).

Inside of the cell, iron can be used for a variety of cellular processes, including hemoglobin synthesis, cell signaling, iron-sulfur cluster group formation, energy production, DNA synthesis, and cell respiration (Abbaspour et al., 2014). Because of it’s potential to form toxic free radicals, iron, that is, not used by the cell or exported, is stored in the iron storage protein ferritin, a nanocage made up of various repeats of light and heavy chains that can oxidize and store up to 5,000 molecules of iron (Plays et al., 2021). The heavy chains are responsible for oxidation of iron prior to storage in the light chains (Plays et al., 2021). When the cell develops an increased need for iron, autophagosomes and autolysosomes are utilized to free ferritin bound iron for use through a nuclear receptor activated 4 (NCOA4) mediated process known as ferritinophagy (Liu et al., 2022). This process is IRP-independent and a secondary pathway through which ferritin is degraded (Liu et al., 2022).

Movement of iron within the cell is achieved by chaperone proteins, such as poly (rC)-binding protein 1 (PCBP1), which allow iron to move throughout the cell without contributing to ROS formation (Patel et al., 2021). In some cells, ferrous iron in the labile iron pool can be transported through the membrane transport protein ferroportin into the plasma, where it is almost immediately bound to transferrin (Sheftel et al., 2012; Chifman et al., 2014). Prior to this export, it must be oxidized back to its ferric form, as discussed previously. The binding of iron to transferrin is possible because of the presence of a carbonate in transferrin that contains a charge opposite that of ferric iron (Ogun and Adeyinka, 2022). This process is essential to allow transferrin to safely move iron throughout the body without forming toxic free radicals (Chifman et al., 2014).

## 4 Ferroptosis

In 2012, Scott Dixon and colleagues in the Stockwell lab described a novel form of regulated cell death they coined ferroptosis due to its dependence on iron availability (Dixon et al., 2012). Ferroptosis occurs as the result of the iron-dependent accumulation of lipid reactive oxygen species (ROS) and results in shrunken mitochondria with thickened membranes (Dixon et al., 2012). The description of ferroptosis as an alternative form of programmed cell death has resulted in a booming new area of research across many chronic diseases including cancer, neurodegeneration, and cardiovascular diseases (Stockwell, 2022). Key to understanding the therapeutic potential of ferroptosis in health and disease is the availability of two agents of ferroptosis induction characterized in the original description of ferroptosis: erastin and RAS-selective lethal 3 (RSL3) (Dixon et al., 2012).

Erastin induces ferroptosis by blocking the function of SLC7A11 resulting in downstream interruption of glutathione (GSH) production, and subsequently GPX4 synthesis (Dixon et al., 2014). RSL3 induces ferroptosis by directly binding to and inhibiting the function of GPX4 (Yang et al., 2014). Ferroptosis then occurs as the result of excess lipid peroxidation, beyond the capacity of the endogenous lipophilic antioxidant glutathione peroxidase 4 (GPX4) to repair them (Stockwell, 2022). The ensuing lipid ROS accumulation leads to altered function and membrane destruction, resulting in cell death (Lee et al., 2021).

GPX4 is an endogenous antioxidant that selectively targets lipids (Lee et al., 2021; Stockwell, 2022). The canonical GPX4 production pathway begins with system Xc^−^, which refers to the two membrane transport proteins solute carrier family 3 member 2 (SLC3A2) and solute carrier family 7 member 11 (SLC7A11) (Stockwell, 2022). These proteins function as antiporters, responsible for the import of cystine and export of glutamate, respectively (Lu et al., 2017). Inside of the cell, cystine is reduced to two cysteines which are incorporated into GSH prior to its oxidation by GPX4 to reduce lipid peroxides to lipid alcohols (Lu et al., 2017; Stockwell, 2022). As such, exogenous lipophilic antioxidants can be used to prevent ferroptosis (Lee et al., 2021). The two most commonly used examples are liproxstatin-1 (Lip-1) and ferrostatin-1 (Fer-1) (Zilka et al., 2017; Stockwell, 2022). Both of these function as radical trapping antioxidants (RTAs), meaning that they prevent autooxidation rather than influencing the activity of the oxidases contributing to lipid peroxide formation (Zilka et al., 2017). Other inhibitors of ferroptosis also exist, for example: probucol, an antioxidant drug used to treat dyslipidemia (Yamashita et al., 2015), is able to inhibit ferroptosis (Stockwell, 2022). Additionally, selenium, nitroxide, iron chelators like deferoxamine (DFO) and even, at very high doses, necrostatin-1, a necrosis inhibitor, have been shown to inhibit ferroptosis (Stockwell, 2022).

Since the generation of lipid ROS is the main mechanism of damage leading to cell death by ferroptosis, lipid metabolism plays a critical role in ferroptosis. Phospholipids that contain polyunsaturated fatty acid (PUFA-PLs) are at an especially high risk for peroxidation, particularly PUFAs containing adrenic or arachidonic acids (Lee et al., 2021; Stockwell, 2022). This role applies specifically to membrane-incorporated PUFA-PLs, as the oxidation of PUFAs that are not membrane anchored and incorporated into PUFA-PLs do not contribute to ferroptosis (Lee et al., 2021). The lipid metabolism protein, achaete-scute family belch transcription factor 4 (ASCL4), which incorporates long chain fatty acids and acyl-CoA into fatty acid esters prior to phospholipid generation by lysophosphatidylcholine acyltransferase 3 (LPCAT3) is an important mediator between lipid metabolism and ferroptosis (Doll et al., 2017). In breast cancer cells, decreased or increased ASCL4 expression is associated with reduced or augmented ferroptosis sensitivity, respectively (Lee et al., 2021). Additionally, monounsaturated fatty acids have been shown to be ferroptosis protective, possibly due to competition with PUFAs for phospholipid synthesis (Lee et al., 2021).

There are two main mechanisms of lipid peroxidation in ferroptosis: enzyme-mediated and auto-oxidation (Lee et al., 2021). Enzymatic oxidation is the result of the action of many iron containing proteins, including lipoxygenases (LOXs), cytochrome P450 oxidoreductase (POR), and nicotinamide adenine dinucleotide phosphate (NADPH) oxidases (NOXs) (Lee et al., 2021). LOXs function by oxidizing PUFAs to PUFA lipid hyperoxides (Kuhn et al., 2015). Increased POR activity is hypothesized to accelerate the conversion of ferrous iron in its heme group to ferric iron and *vice versa*, either promoting or directly contributing to lipid peroxidation (Lee et al., 2021). Finally, NOXs generate lipid superoxides through a reaction in which NADPH and oxygen are converted to a hydrogen, a superoxide radical, and NADP^+^ (Panday et al., 2015). Non-enzymatic auto-oxidation occurs as the result of Fenton chemistry whereby ferrous iron and hydrogen peroxide (H_2_O_2_) react resulting in hydroxyl radicals and ferric iron, amongst other products (Dixon et al., 2012; Lee et al., 2021).

When ferroptosis was originally described, it was demonstrated that iron chelation and supplementation decreased and increased ferroptosis, respectively (Dixon et al., 2012). Thus, the name, ferroptosis, was inspired by the essentiality of available redox-active iron for any of the above-mentioned processes to occur. Li et al. (2017) demonstrated that exposure to excess levels of both hemoglobin and ferrous iron resulted in ferroptosis. The authors showed that excess hemoglobin results in increased lipid peroxide accumulation as a result of GPX4 inhibition (Li et al., 2017). It is important to note that increased total cellular iron is not necessary for ferroptosis, as release of iron from ferritin can be ferroptosis promoting (Quiles del Rey and Mancias, 2019). Despite these findings, the role of iron metabolism in ferroptosis is only beginning to be elucidated and the functions of many proteins involved in iron metabolism in ferroptosis remain poorly understood.

## 5 Iron and IRPs in ferroptosis

Ferroptosis is driven by extensive iron-dependent accumulation of lipid reactive oxygen species (ROS), which ultimately commits cells to death (Dixon et al., 2012). Importantly, IRPs, the principal regulators of cellular iron homeostasis themselves are also regulated by iron availability and reactive oxygen species (Anderson et al., 2012), yet our understanding of how the IRP-IRE-system contributes to iron accumulation during ferroptotic cell death is still in its infancy. Investigations into the roles of IRPs in ferroptosis are made complicated however because even though IRP1 and IRP2 are ubiquitously expressed (Meyron-Holtz et al., 2004a), their relative expression levels differ in cell type and tissue-dependent manners, and they can display distinct biological roles under different physiologic conditions (Meyron-Holtz et al., 2004b).

It is currently understood that uptake of transferrin-bound iron, via the IRP target TFRC, is necessary for ferroptosis and that RNAi knockdown of TFRC decreases ferroptosis sensitivity (Gao et al., 2015). Nevertheless, the question as to why TFRC would continue to import iron following ferroptosis induction at the cost of cell death remains. Research into TFRC regulation during ferroptosis induction has led to conflicting findings. Wang et al. (2016) reported reduced TFRC expression after erastin treatment. Such results are consistent with an appropriate cell response, wherein IRPs sense a relative cellular iron overload and decrease mRNA binding to reduce TFRC expression and subsequent cellular iron uptake (Wang et al., 2016).

Conversely, however, Alvarez et al. (2017) reported increased TFRC expression following erastin treatment. The authors speculated that the increase in TFRC expression is the result of decreased Fe-S biogenesis/stability and the ensuing increase in IRP1 mRNA binding activity (Alvarez et al., 2017). Nonetheless, neither IRP1 nor IRP2 expression or activity were assessed in either of these studies. Given the essentiality of iron availability to the effectiveness of ferroptosis activation, there is a fundamental need to understand the contribution of this major iron regulatory system to ferroptosis to fully harness its therapeutic potential.

IRP2 was first identified as a critical ferroptosis regulatory gene using a high-throughput shRNA screening library in the seminal work by Dixon et al. (2012). A strength of this work was that these findings were then validated by shRNA knockdown of IRP2 and its negative regulatory E3 ubiquitin ligase FBXL5, which resulted in reduced and enhanced sensitivity to ferroptosis induction, respectively. However, IRP2 mRNA binding activity was not assessed, and oxidized IRPs will not bind IRE (Henderson and Kuhn, 1995; Zumbrennen et al., 2009), so it cannot be assumed that increased levels of IRP2 protein expression indicates active IRE binding. Indeed, the effects on downstream IRP2 targets were inconsistent with functional changes in IRP2 mRNA binding activity (Dixon et al., 2012). This raises the possibility that in ferroptosis, IRP2 may be functioning independently of its canonical role in mRNA binding.

**FIGURE 2:**
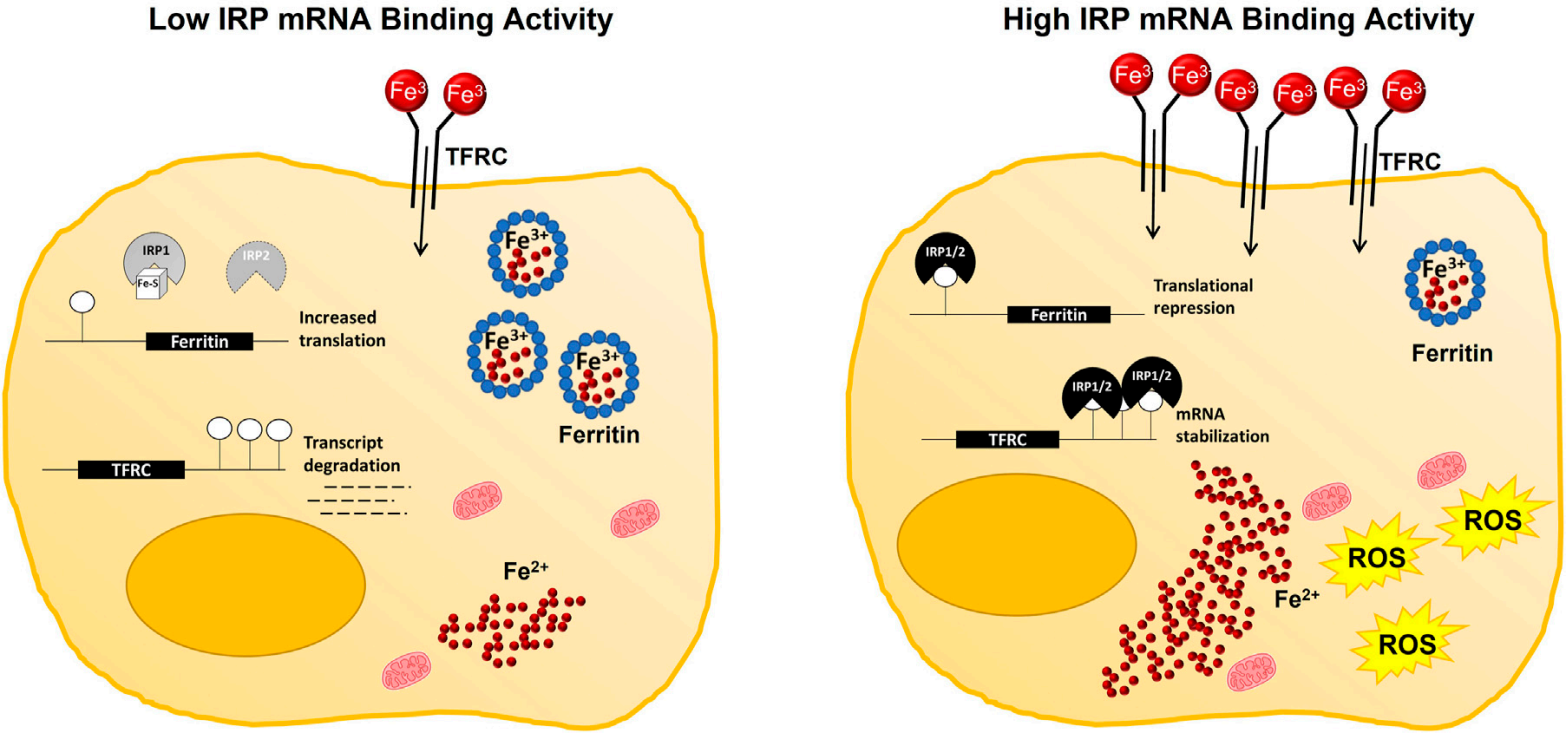
Working model of IRP-mediated contributions to cellular iron availability during ferroptosis. Under iron replete conditions, IRP mRNA binding activity is decreased. Subsequently, iron uptake by TFRC is reduced, while storage of iron in ferritin is increased to maintain a relatively small pool of labile iron within the cell. Whereas, under iron deficient conditions, or with impaired Fe-S cluster biogenesis, IRP mRNA binding activity is increased, reducing the capacity of the cell to safely store iron in ferritin while simultaneously promoting iron uptake by increasing TFRC abundance. Increased IRP mRNA binding activity then facilitates ferroptotic cell death by increasing the size of the labile iron pool and enabling ROS production.

In support of this hypothesis, changes in IRP mRNA binding activity do not always predict differences in ferroptosis sensitivity (Thompson et al., 2020). The context-dependent differences in IRP involvement are likely multifaceted. Factors such as mode of ferroptosis induction (Gryzik et al., 2021), metabolic state of the cell (Novera et al., 2020), and differences in endogenous antioxidant capacities (Cardona et al., 2022) that influence the ability to handle a given amount of labile iron could all contribute to IRP responsiveness. Regardless, increased IRP-IRE binding activity (Chen et al., 2020), and even the increased expression of either IRP1 (Yao et al., 2021; Zhang et al., 2022) or IRP2 (Li et al., 2020) alone have been shown to augment ferroptosis sensitivity (Figure 2).

Fe-S cluster biogenesis is also another critical link between IRP-dependent control of cellular iron homeostasis and ferroptotic cell death as both cysteine deprivation and glutathione depletion promote ferroptosis and diminish Fe-S cluster biogenesis (Sipos et al., 2002; Novera et al., 2020). The formation, or lack thereof then, of an Fe-S cluster into IRP1 determines its role within the cell. When intracellular iron levels are low, IRP1 regulates iron homeostasis through its mRNA binding activity, but under iron adequate conditions, IRP1 primarily exists in an Fe-S cluster containing enzymatic form (Meyron-Holtz et al., 2004a).

The most common means of inducing ferroptosis include inhibiting system xc^−^, and thus preventing cysteine import and glutathione production, or directly inhibiting GPX4 activity. As such, a negative impact on Fe-S cluster biogenesis, and subsequent promotion of IRP1 mRNA binding activity has been assumed by a number of investigators (Yuan et al., 2016; Terzi et al., 2021; Zhang et al., 2022), but has only been indirectly assessed by measuring changes in aconitase activity (Novera et al., 2020; Yao et al., 2021). The accumulation of mitochondrial iron however is also consistent with the hypothesis that ferroptosis induction disrupts Fe-S biogenesis and promotes IRP1 mRNA binding activity (Sipos et al., 2002; Yuan et al., 2016; Novera et al., 2020). Moreover, stabilization of the mitochondrial membrane protein, CDGSH iron sulfur domain 1 (CISD1), which can aid in the repair of oxidatively damaged IRP1 Fe-S clusters, has been shown to inhibit ferroptosis by decreasing mitochondrial lipid peroxidation (Yuan et al., 2016). Thus, current evidence does at least support a role for IRP1 mRNA binding activity in contributing to ferroptotic cell death, but it appears to be due to a pathologic disturbance of Fe-S biosynthesis rather than a response to changes in cellular iron.

It has also been hypothesized that IRP1 expression can be modulated to avoid cell death by ferroptosis (Zhang et al., 2022). Zhang et al. (2022) found that enolase 1 (ENO1), an enzyme involved in glycolysis is able to bind IRP1 mRNA and recruit CCR4-NOT transcription complex subunit 6 (CNOT6), a protein that utilizes its nuclease domain to degrade IRP1 mRNA (Zhang et al., 2022). The authors of the study also reported that expression of solute carrier family 25 member 37 (SLC25A37), also referred to as mitoferrin-1 (MFRN1), the protein responsible for import of iron into the mitochondria, is decreased in response to ENO1 (Zhang et al., 2022). Additionally, they showed that decreased SLC25A37 expression led to decreased ROS formation, leading to the hypothesis that ENO1 regulated an IRP1-SLC25A37 axis to alter ferroptosis sensitivity (Zhang et al., 2022). These findings support another role for IRP1 in ferroptosis.

Intriguingly however, disruption of Fe-S cluster biogenesis by cysteine deprivation in ovarian clear cell carcinoma cell lines only led to ferroptotic cell death in cells which were relying more heavily on glycolysis for energy production (Novera et al., 2020). Whereas cells that were depending more heavily on oxidative phosphorylation appeared to succumb to apoptosis (Novera et al., 2020). The authors postulated the observed differences in cell death may be due to the high use of Fe-S containing proteins to complete oxidative metabolism, and thus metabolic state may be important to consider when assessing the role of IRPs in ferroptosis (Novera et al., 2020). Given the preferences for glycolytic energy production in many tumor cell types, these findings suggest disruption of Fe-S cluster assembly and perturbation of IRP function could be used to further augment ferroptosis sensitivity cancer.

Previous work indicates that in addition to the control of IRP1 function, IRP2 stability is also dependent upon Fe-S cluster assembly proteins (Stehling et al., 2013), but this has only recently been explored in the context of ferroptosis (Terzi et al., 2021). In both reports though, IRP2 stability and mRNA binding activity were increased upon inhibition of cytosolic Fe-S protein assembly. This suggests that activation of ferroptosis by restricting cysteine and glutathione availability would also mimic an iron starvation response by increasing the mRNA binding activity of both IRP1 and IRP2. However, the mRNA binding activity of neither IRP1 nor IRP2 has been fully characterized following treatment with traditional ferroptosis inducing agents such as erastin or RSL3.

Some artemisinin derivatives like artemether (ART) and dihydroartemisinin (DAT) can also be ferroptosis inductive (Chen et al., 2020; Li et al., 2020), and one way these compounds may promote ferroptosis is by increasing IRP mRNA binding activity (Chen et al., 2020; Li et al., 2020). In 2020, when studying ART as a potential liver fibrosis treatment, Li et al. (2020) found that ART induced ferroptosis through IRP2. They reported a dose dependent increase in IRP2 expression in response to ART treatment and that IRP2 knockdown significantly decreased ART’s ability to induce ferroptosis (Li et al., 2020). Intriguingly, labile iron within the cell may also bind directly to DAT. In this form, the DAT-iron complex retains iron’s redox potential but is unable to alter IRP activity (Chen et al., 2020). Thus, artemisinin derivatives may be particularly useful in combination with other small molecule inducers of ferroptosis.

## 6 IRPs in cancer and ferroptosis

In cancer, IRP signaling can be corrupted in an effort to acquire sufficient iron to support rapid cell proliferation. For example, IRP2 overexpression in breast cancer results in increased TFRC expression, decreased ferritin expression, and subsequently an increased labile iron pool (Wang et al., 2014). Increased expression of TFRC was also found to have worse clinical prognosis in patients that had renal cell carcinoma (Greene et al., 2017). As mentioned above, increased expression of TFRC is typically mediated by increased IRP mRNA binding activity, but overexpression of IRP1 was actually found to decrease tumor growth *in vivo* (Chen et al., 2007). Thus, despite their similar roles in the maintenance of iron homeostasis, IRP1 and IRP2 exhibit opposing phenotypes in the reduction and promotion of tumor growth, respectively.

The disparate effects of IRP1 *versus* IRP2 expression in cancer outcomes may be partially explained by the specific 73 amino acid insert in IRP2 that structurally distinguishes it from IRP1. Indeed, overexpression of wild-type IRP2 significantly increased tumor burden in a mouse xenograft model, but when a mutant version IRP2 lacking the 73 amino acid insert was overexpressed in the same model, this response was blunted (Maffettone et al., 2010). Intriguingly, the expression of canonical IRP targets was largely unaffected in tumors expressing either the wild-type or mutant version of IRP, but rather wild-type IRP2 bearing tumors displayed increased levels of MYC proto-oncogene, bHLH transcription factor (MYC) and mitogen-activated protein kinase 1/3 (MAPK1/3) phosphorylation. These findings suggest that IRP2 may promote tumor development independently of its role in iron metabolism.

IRP2 has also been implicated in tumor progression via its capacity to suppress translation of the tumor suppressor gene, tumor protein p53 (TP53) (Zhang et al., 2017). However, this regulation seems to function in a highly regulated feedback loop as TP53 inactivation of the IRE-IRP system can also facilitate tumor cell growth arrest by restricting cellular iron availability (Zhang et al., 2008). This work was recently expanded upon by the discovery that wild-type TP53 can specifically modulate IRP1 RNA binding activity via the transcriptional regulation of the iron-sulfur cluster assembly enzyme (ISCU) (Funauchi et al., 2015). The importance of the iron-TP53 feedback loop in tumor suppression is further supported by the findings that decreased ISCU expression in human liver cancer tissues is associated with TP53 mutations (Funauchi et al., 2015).

As TP53 is the most commonly mutated gene in all of human cancers, our lab then asked the question as to how IRP1 and IRP2 are regulated in cancer cells harboring distinct TP53 mutation types. We found that induction of mutant TP53 expression significantly reduced ferredoxin reductase (FDXR) expression, and that this reduced expression was associated with impaired mitochondrial Fe-S cluster biogenesis and altered IRP function in response to changes in cellular iron availability (Clarke et al., 2019). Notably, proper FDXR signaling has also been shown to be essential for IRP2 mediated control of TP53-dependent tumor suppression (Zhang et al., 2017). In humans, FDXR is critical for Fe-S cluster biogenesis and its reduction is associated with misregulation of cellular iron homeostasis (Shi et al., 2012). As such, ferroptosis induction has been proposed as a way to therapeutically target tumor cells expressing distinct mutant TP53 subtypes (Thompson et al., 2020).

Fe-S cluster containing proteins are also essential components of energy metabolism and DNA repair enzymes, and their impaired assembly could significantly impact tumor progression. Intriguingly, the antidiabetic drug pioglitazone was recently shown to inhibit iron transfer into the mitochondria by stabilizing the [2Fe-2S] cluster in CDGSH iron sulfur domain 1 (CISD1) (Zuris et al., 2011). It was then proposed that an unrecognized benefit of pioglitazone use for diabetic patients might be reduced ROS production as a result of decreased mitochondrial iron availability. However, an unintended consequence of this mitochondrial iron restriction could be diminished ferroptosis sensitivity. Indeed, Yuan et al. (2016) demonstrated that pioglitozone diminishes ferroptotic cell death in a CISD1-dependent manner by protecting against mitochondrial iron accumulation. Continued investigations are needed to delineate how pioglitazone influences cellular IRP mRNA binding activity, and how this influence could impact ferroptosis sensitivity.

## 7 Conclusion

Cancer cells are extravagant users of iron, and as such, much effort has been devoted to taking advantage of cancers cells’ “iron addiction” by restricting iron availability (Lui et al., 2015). However, these approaches are confounded by the essential nature of iron for noncancerous cells as well. Ferroptosis has been described as a novel approach to exploiting the toxic nature of iron to promote programmed cell death, but again the toxic potential of iron for all cell types must be considered. Given the essentiality of the IRP-IRE system to the maintenance of iron homeostasis, and the growing body of evidence implicating the key players of this system in ferroptotic cell death, delineating the specific roles of IRP1 and IRP2 in ferroptosis is of fundamental importance to fully harness its chemotherapeutic potential.
